# Novel Therapeutic Effects in Rat Spinal Cord Injuries: Recovery of the Definitive and Early Spinal Cord Injury by the Administration of Pentadecapeptide BPC 157 Therapy

**DOI:** 10.3390/cimb44050130

**Published:** 2022-04-27

**Authors:** Darko Perovic, Marija Milavic, Stjepan Dokuzovic, Ivan Krezic, Slaven Gojkovic, Hrvoje Vranes, Igor Bebek, Vide Bilic, Nenad Somun, Ivan Brizic, Ivan Skorak, Klaudija Hriberski, Suncana Sikiric, Eva Lovric, Sanja Strbe, Milovan Kubat, Alenka Boban Blagaic, Anita Skrtic, Sven Seiwerth, Predrag Sikiric

**Affiliations:** 1 Department of Pharmacology, School of Medicine, University of Zagreb, 10000 Zagreb, Croatia; dperovic@kbd.hr (D.P.); s.dokuzovic07@gmail.com (S.D.); ivankrezic94@gmail.com (I.K.); slaven.gojkovic.007@gmail.com (S.G.); hrvoje.vranes@gmail.com (H.V.); ibebek@yahoo.com (I.B.); nenad.somun1@gmail.com (N.S.); 2360999@gmail.com (I.B.); ivan.skorak@gmail.com (I.S.); klaudija.hriberski@gmail.com (K.H.); strbes@gmail.com (S.S.); abblagaic@mef.hr (A.B.B.); 2 Department of Surgery, Clinical Hospital Dubrava, 10000 Zagreb, Croatia; 3 Department of Pathology, School of Medicine, University of Zagreb, 10000 Zagreb, Croatia; marija.milavic@mef.hr (M.M.); suncana.sikiric@mef.hr (S.S.); eva.lovric@kb-merkur.hr (E.L.); sven.seiwerth@mef.hr (S.S.); 4 Clinical Hospital of Traumatology, Sestre Milosrdnice University Hospital Center, 10000 Zagreb, Croatia; vide.bilic@gmail.com; 5 Department of Forensic Medicine and Criminology, School of Medicine, 10000 Zagreb, Croatia; milovan.kubat@mef.hr

**Keywords:** pentadecapeptide BPC 157, spinal cord early injury, spinal cord definitive injury, recovery, therapy

## Abstract

Recently, marked therapeutic effects pertaining to the recovery of injured rat spinal cords (1 min compression injury of the sacrocaudal spinal cord (S2-Co1) resulting in tail paralysis) appeared after a single intraperitoneal administration of the stable gastric pentadecapeptide BPC 157 at 10 min post-injury. Besides the demonstrated rapid and sustained recovery (1 year), we showed the particular points of the immediate effect of the BPC 157 therapy that began rapidly after its administration, (i) soon after injury (10 min), or (ii) later (4 days), in the rats with a definitive spinal cord injury. Specifically, in counteracting spinal cord hematoma and swelling, (i) in rats that had undergone acute spinal cord injury, followed by intraperitoneal BPC 157 application at 10 min, we focused on the first 10–30 min post-injury period (assessment of gross, microscopic, and gene expression changes). Taking day 4 post-injury as the definitive injury, (ii) we focused on the immediate effects after the BPC 157 intragastric application over 20 min of the post-therapy period. Comparable long-time recovery was noted in treated rats which had definitive tail paralysis: (iii) the therapy was continuously given per orally in drinking water, beginning at day 4 after injury and lasting one month after injury. BPC 157 rats presented only discrete edema and minimal hemorrhage and increased *Nos1*, *Nos2*, and *Nos3* values (30 min post-injury, (i)) or only mild hemorrhage, and only discrete vacuolation of tissue (day 4, (ii)). In the day 4–30 post-injury study (iii), BPC 157 rats rapidly presented tail function recovery, and no demyelination process (Luxol fast blue staining).

## 1. Introduction

This study further centered the stable gastric pentadecapeptide BPC 157 therapy in rats with a spinal cord injury [1] to show that it might be even more suited for a practical therapy resolution. Namely, the recent therapeutic recovery demonstration in the injured rats (evident functional, microscopic, and electrophysiologic recovery, daily, weekly, monthly, and one year) needed to be further supplemented. With an intraperitoneal application, the presented efforts of the BPC 157-induced recovery included 1 min of compression injury of the sacrocaudal spinal cord (S2-Co1), definitive tail paralysis, and a huge hemorrhagic zone in the lateral and posterior white columns (the grey matter had been spared). Of note, the therapeutic effect of BPC 157 was promising since it took place at 10 min post-compression injury, in the already advanced reperfusion phase (note, in the spinal cord injured rats, there was a permanent reperfusion), and it was uninterrupted as no spontaneous spinal cord injury-induced disturbances reappeared [1]. However, the recovery was still complex following a single intraperitoneal administration at 10 min post-injury [1] as a particular point relatively soon after the injury induction. Further research should clarify the prime effect (immediate effect of the intraperitoneal administration at 10 min post-injury) and the practical application (at the fourth post-injury day, the immediate effect of the intragastric application; the therapy given continuously per orally in drinking water).

Thus, to clarify the rapid and sustained recovery [1], spinal cord hematoma, and swelling, we further substantiated how the prompt effect of the BPC 157 therapy started immediately after its administration, challenged either in the early course or in the later course of the spinal cord injury. Firstly, the BPC 157 therapy was administered intraperitoneally soon after the injury (10 min) [1]. We focused on the subsequent immediate period: the first 10 min–30 min post-injury period (assessment of gross, microscopic, and gene expression changes). Moreover, at the fourth post-injury day, we further substantiated that an equipotent effect of the BPC 157 therapy, given as an intragastric bolus, could occur even when given at later time to the rats with a definitive spinal cord injury. Therefore, in the rats with a protracted spinal cord injury, we focused on the immediate rescuing effects of the treatment over a 20 min period upon receiving the treatment. The attenuation of the spinal cord hematoma and swelling may be a common beneficial effect from both early and delayed treatment application. At that point, on the fourth post-injury day, the additional practical significance of BPC 157 therapy was demonstrated as the therapy was given continuously per orally in drinking water to the rats who already had an advanced spinal cord injury, until the rats were sacrificed on day 30 after injury. Of note, a BPC 157 regimen given in drinking water has consistently been shown to be effective in previous studies (for a review, see [2,3,4,5,6,7,8,9,10,11,12,13,14,15,16,17,18,19,20]).

Furthermore, BPC 157 as a native gastric pentadecapeptide which does not have a lethal dose (LD1) has a profound cytoprotective activity, as has been demonstrated in ulcerative colitis trials (for a review, see [6,18,19]). The fact that it is not degraded in human gastric juice for more than 24 h demonstrates the effectiveness of a therapeutic per oral regimen (for a review, see [18]).

In addition, there is ample evidence supporting the claim that BPC 157 therapy might also be consistently effective in spinal cord injuries [1].

The anti-inflammatory activity, capsaicin-induced somatosensory neurons damage counteraction [21], recovery of sciatic nerve after transection [22], and cultured enteric neurons and glial cells protection [23] have been all reported. A particular neuroprotective effect occurred in the therapy of the retinal injury and glaucoma rat models [24,25]. Pentadecapeptide BPC 157 also ameliorated concussive trauma-induced brain injury [26]. Severe encephalopathies ameliorated by BPC 157 therapy were induced by various noxious procedures [27,28,29,30,31,32,33,34]. These were the non-steroidal anti-inflammatory drugs (NSAIDs) overdose [27,28,29,30,31], insulin overdose seizures [32], neurotoxin cuprizone-induced multiple sclerosis in a rat model [33], and magnesium overdose [34], all as mechanisms of imparting encephalopathies in rats. Importantly, the therapy also included the consequences thereof (i.e., gastrointestinal and/or liver lesions [27,28,29,30,31,32] as well as severe muscle weakness [33,34]). Interestingly, as a part of its innate particular cytoprotective activity, the BPC 157-induced direct endothelium protection [35] functions throughout rapidly activated alternative bypassing pathways [25,36,37,38,39,40,41,42,43,44]. Consequently, the peripheral and central vascular occlusion disturbances were alleviated [25,36,37,38,39,40,41,42,43,44]. The severe venous occlusion- and arterial occlusion-induced syndromes [25,36,37,38,39,40,41,42] as well as occlusion-like syndromes following major intoxication (alcohol, lithium), maintained intra-abdominal hypertension [43,44,45], and isoprenaline-induced myocardial infarction [46] were all counteracted as well. Thus, in view of the venous occlusion caused by the spinal cord compression [47], it is conceivable that the reestablishment of blood flow upon treatment may certainly contribute to the rapid recovery effect noted [1]. Illustratively, when given during reperfusion as was also the case with the spinal cord injury therapy [1], BPC 157 counteracted the development of the stroke caused by the bilateral clamping of the common carotid arteries [48]. Sustained brain neuronal damages were resolved in the injured rats as well as the disturbed memory, locomotion, and coordination [48]. This therapeutic effect was supported by a particular gene expression in the hippocampal tissues that appeared in BPC 157-treated rats [48].

Pentadecapeptide BPC 157 also induces the healing of severely injured muscles (i.e., complete transection, crush, and denervation injuries [49,50,51,52]). In the counteraction of the effects of the intramuscular succinylcholine application [53], muscle healing always coincides with the recovered muscle function, the recovery of neuromuscular junction lesions, and the counteraction of the fasciculations, paralysis, and hyperalgesia [53]. The interaction of BPC 157 with various molecular pathways [3,4,38,48,54,55,56,57,58,59] enables its activity in the counteraction of the increased levels of pro-inflammatory and pro-cachectic cytokines and of downstream pathways, and the reversal of tumor-induced muscle cachexia [3], while also facilitating its healing effect as a membrane stabilizer (counteracted leaky gut syndrome) [4] and a free radical scavenger [60,61,62]. Finally, BPC 157 was also recently shown to have intrinsic antidotal activity against the adverse effects of lidocaine and local anesthetics [63] (in particular, limb function failure (spinal (L4–L5) intrathecal block, bradycardia and tonic-clonic convulsions, depolarization of the HEK293 cell) [63]. On the other hand, since an immediate hemorrhage leads to the death of neurons and oligodendrocytes [64,65,66,67,68], BPC 157 may have a useful hemostatic effect [38,69,70,71,72] to attenuate a spinal cord injury.

Thus, BPC 157 counteracts axonal and neuronal necrosis, demyelination, and cyst formation, providing evidence for its role in the functional rescue of the paralyzed tail [1]. The possibility that BPC 157 therapy might affect many mechanisms of secondary neuronal injury [1] was supported by the immediate events occurring upon BPC 157 application at 10 min post-injury, as well as at day 4 after injury. Thereafter, the practical applicability was supported by the per oral application of the therapy given continuously in drinking water until the end of the one-month period. Gene expression analysis included the mechanistic target of rapamycin (*Mtor*), vascular endothelial growth factor A (*VEGFa*), protein tyrosine kinase 2 (*Pkt2*), prostaglandin-endoperoxide synthase 2, (*Ptgs2*, cyclooxygenase (Cox) 2), and nitric oxide synthase (NOS) (*Nos1*, *Nos2*, *Nos3*), commonly implicated in the injured spinal cord [73,74,75,76,77,78,79,80]. At present, BPC 157 is known to largely interact with prostaglandin-, nitric oxide (NO)-, and the vascular endothelial growth factor (VEGF)-system in a particular way (for a review, see [13,14,18,54,58]). Thereby, this may reveal a specific background for the beneficial effects of BPC 157, providing that the gene expression indicates a precisely coordinated series of events.

## 2. Materials and Methods

### 2.1. Animals

Wistar Albino male rats (aged 12 weeks, 350–400 g b.w.), in-house bred, (animal facility Pharmacology—School of Medicine, Zagreb Croatia, registered by the Directorate of Veterinary Studies; Reg. No: HR-POK-007), were acclimated for 5 days and randomly assigned to the experiments (6 animals at least per each experimental group and interval), all of which were approved by the Local Ethics Committee. Laboratory animals were housed in PC cages in conventional laboratory conditions at a temperature of 20–24 °C, a relative humidity of 40–70%, and a noise level of 60 DCB. Each cage was identified following the date, study number, group, dose, number, and sex of each animal. Fluorescent lighting provided illumination for 12 h per day. A standard GLP diet and fresh water were provided *ad libitum*. Furthermore, all experiments were carried out under blind protocols and the effect was assessed by examiners who were completely unaware of the given protocol. We certified that the government regulation concerning the ethical use of animals during this research was adhered to.

### 2.2. Drugs

Pentadecapeptide BPC 157 (GEPPPGKPADDAGLV, M.W. 1419), (Diagen, Ljubljana, Slovenia) dissolved in saline was used in all experiments [1]. The peptide BPC 157 is part of the sequence of the human gastric juice protein BPC and is freely soluble in water and saline at pH 7.0. It was prepared as described previously with 99% high-pressure liquid chromatography (HPLC) purity, expressing 1-des-Gly peptide as an impurity [1].

### 2.3. Surgery and Spinal Cord Injury

As described previously [1], deeply anesthetized (3% isoflurane, ketamine 50 mg/kg b.w.) rats were subjected to a laminectomy at the lumbar level L2–L3, which corresponds to the sacrocaudal spinal cord S2-Co1. A neurosurgical piston graduated to 60–66 g was placed over the exposed dura and left for 60 s for a compressive injury.

We envisaged two particular points, early and late, for the therapy application. (i). The point of the 10 min post-injury was used for the estimation of the immediate changes in the very early course of the spinal cord injury. (ii; iii). Post-injury day 4 was chosen for the estimation of the immediate changes upon the given therapy in the already advanced course of the spinal cord injury (ii) and as the starting point for the initiation of the per oral therapy continuously given per orally in drinking water (iii).

#### 2.3.1. Early Post-Injury Course, Immediate Effect

(i). To observe an immediate effect, after the piston removal a single intraperitoneal injection of either saline (5 mL/kg b.w.) or BPC 157 (2 μg/kg b.w.) was administered at 10 min post-injury. The spinal cords were filmed under a microcamera for 20 min, and then the rats were sacrificed (i.e., 30 min after injury).

#### 2.3.2. Delayed Post-Injury Course, Immediate Effect

(ii). Alternatively, after the piston removal, muscle and skin incisions were closed. On the post-injury day 4, the rats were reoperated on and under deep anesthesia, the injured spinal cord was exposed, and one single intragastric application of either saline (5 mL/kg b.w.) or BPC 157 (10 ng/kg b.w.) was administered. Again, the spinal cords were filmed under a microcamera for 20 min and thereafter, the rats were sacrificed.

#### 2.3.3. Delayed Post-Injury Course, Long-Term Effect

(iii). For the observation of a long-term effect of delayed treatment, in rats with definitive tail paralysis (on postoperative day 4), therapy was begun and continuously given per orally in drinking water (water 12 mL/rat/day; BPC 157 10 μg/kg b.w., 0.16 μg/mL). Thus, this therapy protocol spanned the period from day 4 after injury induction until the sacrifice at one month following injury.

In addition, a group of untreated rats were sacrificed to establish the initial histological injury presentation on day 1 (10 min after a spinal injury) (i) as well as on day 4 (ii; iii).

### 2.4. Clinical Evaluation

We employed the same scoring as in a prior study [1]. On day 4 post-injury, before therapy and daily until day 30 post-injury, we scored tail motor function as follows (score 0–5): 0—autotomy; 1—complete loss of tail function; 2—elevation maximum of 1/4 of tail length; 3—elevation maximum of 1/2 of tail; 4—elevation maximum of 3/4 of tail length; 5—normal function. Likewise, tails were observed for spasticity after manual stimulation with the standardized stretch/rub maneuver and scored according to the Bennett scale (2): 0—normal; 1—tail flaccid; 2—flexor muscle hypertonus; tail coiled and stiff; 3—hyperreflexia, e.g., coiling flexor spasm and clonus in response to light touch or stretch; 4—hypertonus in flexor and extensor muscles, clonus and hyperflexia, the latter including a positive curling reaction.

### 2.5. Histology

A 10-mm long piece of the spinal column (the L2–L3 segment) and the surrounding muscle tissue was collected from each sacrificed animal and fixed in phosphate-buffered 4% formaldehyde (pH 7.4). After fixation, the spinal column was decalcinated and embedded in paraffin. Serial 5-μm cross sections were made, and stained with hematoxylin/eosin (Kemika, Zagreb, Croatia) and Luxol fast blue (Biognost^®^, Zagreb, Croatia). Part of the spinal cord grey and white matter was used for analysis under light microscopy (magnification × 300). According to previous studies [1], the intensity and distribution of the following pathological spinal cord changes were evaluated semiquantitatively (0—no changes; 1—small or focal changes; 2—moderate changes; 3—numerous confluent changes): (a) the hemorrhagic zone, (b) edema, (c) vacuolation of tissue matter, (d) the loss of neurons in anterior horn and intermediate grey matter, and (e) the loss of lateral and posterior spinal column tracts. Histochemical staining with Luxol fast blue was undertaken to analyze the myelin fibres of the posterior and lateral spinal column tracts, along with the anterior horn, and the intermediate grey matter.

### 2.6. Spinal Cord Volume Presentation Proportional with the Change in the Spinal Cord Surface Area

For the spinal cord volume/surface area correlation, we used the method previously described in [38,39,40,41,42,43,44,45,46]. The presentation of the spinal cord was recorded in deeply anesthetized rats, with a camera attached to a VMS-004 Discovery Deluxe USB microscope (Veho, Denver, CO, USA), before the procedure in normal, healthy rats (before injury induction) and then, in spinal cord-injured rats, before therapy application at 10 min or at day 4 after injury, and then at 1 min, 5 min, 15 min, and 20 min intervals after therapy application, before the animals were sacrificed. In each photograph, the border of the spinal cord (or spinal cord hematoma) was marked using ImageJ computer software and then the surface area (in pixels) of the spinal cord (or spinal cord hematoma) was measured using a measuring function. This was undertaken with spinal cord photographs before the application and at intervals after the application for both the control and treated animals. The spinal cord area before the procedure and application (assessment at day 1 (i)) (or spinal cord assessment at day 4 (ii)) and spinal cord hematoma before application (assessment at 10 min (i) and day 4 (ii) post-injury) was marked as 100% and the ratio of each subsequent spinal cord area to the first area was calculated ($\frac{A_{2}}{A_{1}}$). Starting from the square-cube law in Equations (1) and (2), an equation for the change in spinal cord volume proportional to the change in the spinal cord surface area Equation (6) was derived. In Equations (1)–(5) *l* is defined as any arbitrary one-dimensional length of spinal cord segment used only for defining the one dimensional proportion (*l*_2_*/l*_1_) between two observed spinal cords and as an inter-factor (and because of that Equation (6) was not measured) for deriving the final expression Equation (6). The procedure was as follows:

(1) $$A_{2}=A_{1}\times {\left(\frac{l_{2}}{l_{1}}\right)}^{2}$$

(Square-cube law),

(2) $$V_{2}=V_{1}\times {\left(\frac{l_{2}}{l_{1}}\right)}^{3}$$

(Square-cube law),

(3) $$\frac{A_{2}}{A_{1}}={\left(\frac{l_{2}}{l_{1}}\right)}^{2}$$

(From Equation (1), after dividing both sides by *A*_1_),

(4) $$\frac{l_{2}}{l_{1}}=\sqrt{\frac{A_{2}}{A_{1}}}$$

(From Equation (3), after taking the square root of both sides),

(5) $$\frac{V_{2}}{V_{1}}={\left(\frac{l_{2}}{l_{1}}\right)}^{3}$$

(From Equation (2), after dividing both sides by *V*_1_),

(6) $$\frac{V_{2}}{V_{1}}={\left(\sqrt{\frac{A_{2}}{A_{1}}}\;\right)}^{3}$$

(After incorporating the expression from Equation (4) into Equation (5))

### 2.7. Gene Expression Analysis

After BPC 157 application, observation under a microcamera, and the sacrifice of animals, the tissue was rapidly dissected, snap-frozen in liquid nitrogen, and stored at −80 °C until RNA extraction. The tissue was homogenized using the Bio-Gen PRO200 homogenizer (PRO Scientific, Oxford, Connecticut, Oxford, CT, USA) in 1000 μL of TRIzol (Invitrogen, Thermo Fisher Scientific, Waltham, MA, USA) and RNA extraction was performed using a TRIzol-based reagent method according to the manufacturer’s instructions.

After RNA extraction, quantification was performed with a Nano Drop ND-1000 spectrophotometer (Nano DropTechnologies, Thermo Fisher Scientific, Waltham, MA, USA). A High Capacity cDNA Reverse Transcription Kit (Applied Biosystems, Thermo Fisher Scientific, Waltham, MA, USA) was used to perform the reverse transcription following the manufacturer’s instructions and using the ProFlex PCR System machine (Applied Biosystems, Thermo Fisher Scientific, Waltham, MA, USA). The TaqMan Gene Expression Assay (Applied Biosystems, Termo Fisher Scientific, Waltham, MA, USA) with the TaqMan Gene Expression Master Mix were used for the gene expression analysis of selected genes (Table 1). A quantitative PCR was carried out in duplicate for every sample. The Cobas z 480 instrument (Hoffmann-La Roche Ltd., Basel, Switzerland) was used to perform the qPCR according to the following protocol: 2 min at 50 °C, 10 min at 95 °C, 45 cycles of 15 s at 95 °C, and 1 min at 60 °C.

*Actb* was chosen as the reference gene for the normalization of the *Akt1*, *Nos1*, *Nos2*, *Nos3*, *Mtor*, *Prkcg*, *Ptgs2*, *Ptk2*,and *Vegfa* gene expression data (Table 1).

The difference in gene expression between treated and non-treated samples was analyzed using the formula 2^−ΔΔCt^, where ΔΔCt is the difference between the ΔCt of the treated sample and the ΔCt of the non-treated sample.

### 2.8. Statistical Analyses

Scored data were expressed as minimum, median, maximum (Min/Med/Max), and compared using the Kruskal–Wallis ANOVA test (*p*-values < 0.05 were considered significant) followed by the Mann–Whitney U test (*p*-values < 0.025 were considered significant—Bonferroni correction). Numeric data were expressed as mean ± SD and compared with the one-way ANOVA followed by the LSD test. The statistical program (Statistica for Windows ver. 5.0, StatSoft Inc. Tulsa, OK, USA) was used for the statistical analysis. *p*-values < 0.05 were considered significant.

## 3. Results

### 3.1. Clinical Examinations

#### 3.1.1. Tail Motor Function Score

The post-injury tail motor function score generally failed in spinal cord-injured rats who only received drinking water as therapy.

**Figure 1:**
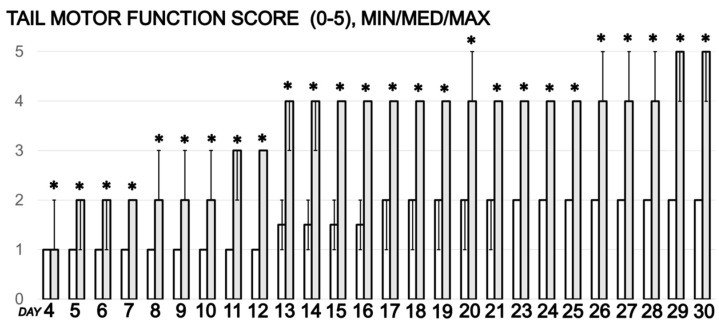
In rats that underwent spinal cord injury, debilitated (control rats drinking water 12 mL/day/rat, white bars) and rescued tail motor function (BPC 157 (10 μg/kg) in drinking water since post-injury day 4, grey bars) presented by tail motor function score, Min/Med/Max. * *p* < 0.05, at least, vs. control.

Conversely, per oral BPC 157 administration resulted in the recovery of the rats even when given at 4 days post-injury, when permanent damage is expected to be established. After initial incapacity, all treated rats showed an improved motor function (Figure 1).

**Figure 2:**
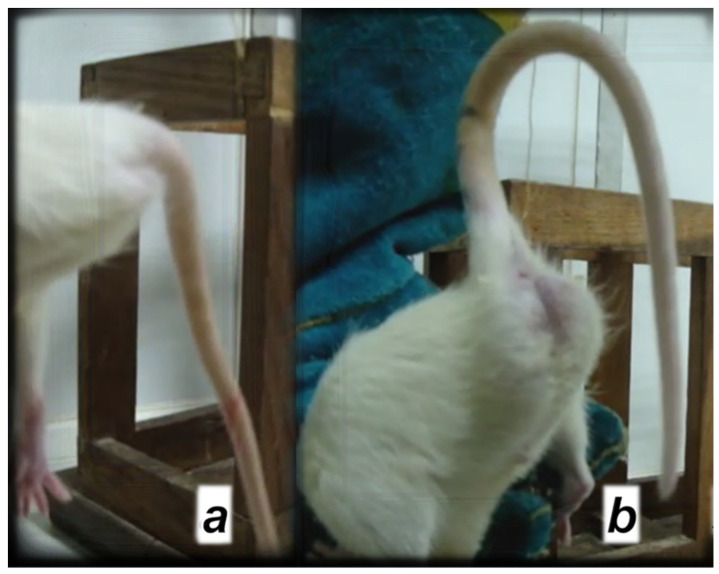
Illustrative presentation of (**a**) a debilitated tail motor function in control rats with a spinal cord injury and received drinking water 12 mL/day/rat; (**b**) rescued tail motor function in rats with a spinal cord injury and received BPC 157 (10 μg/kg) in drinking water from day 4 post-injury. Post-injury day 30.

Figure 2 illustrates the presentation of the tail motor function full recovery in the spinal cord-injured rats that received BPC 157 (10 μg/kg) in drinking water from day 4 post-injury.

#### 3.1.2. Tail Spasticity

**Figure 3:**
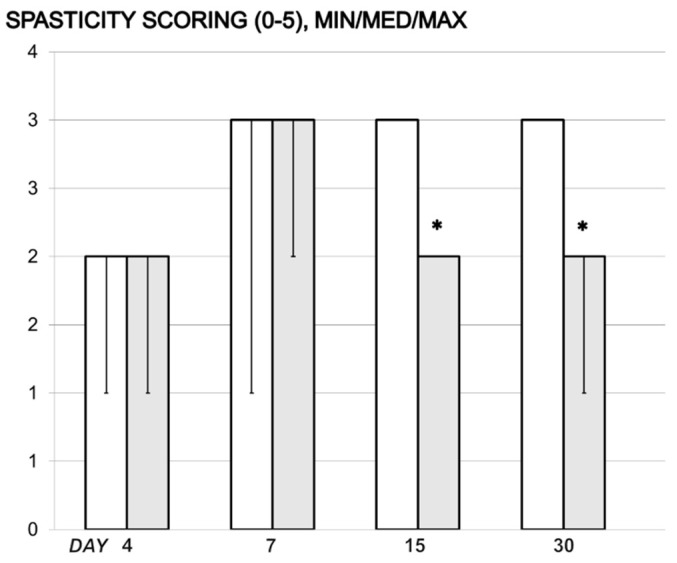
In rats with a spinal cord injury, debilitated (control rats drinking water 12 mL/day/rat, white bars) and rescued tail spasticity (BPC 157 (10 μg/kg) in drinking water from day 4 post-injury, grey bars) presented by the tail spasticity score, Min/Med/Max. * *p* < 0.05, at least, vs. control.

Unlike constant spasticity in the controls, this was counteracted in BPC 157-treated rats, resulting in no spasticity from day 15 onward (Figure 3).

#### 3.1.3. Gross Assessment of the Spinal Cord Injury Hematoma and Swelling

**Figure 4:**
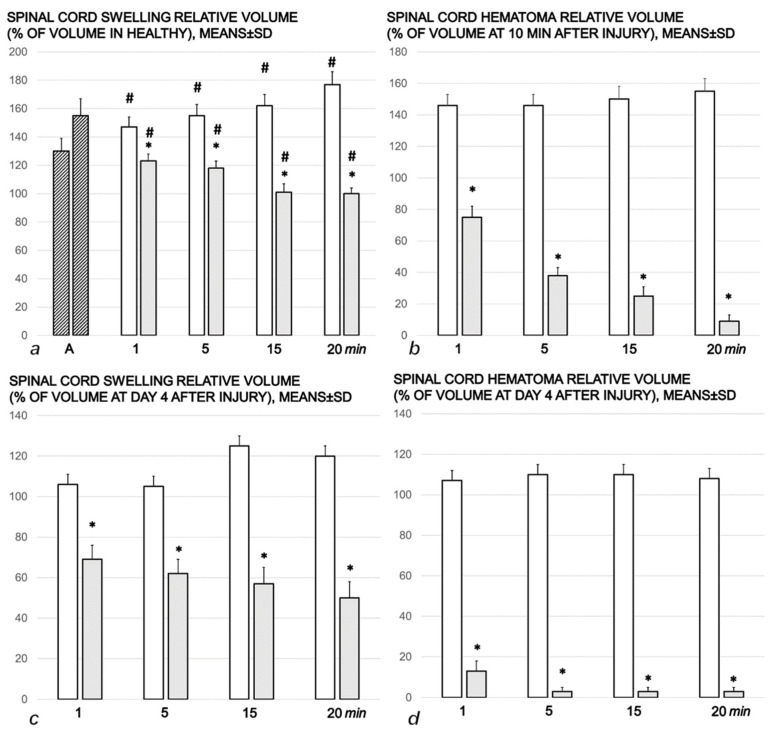
Relative volume of spinal cord swelling and hematoma. Spinal cord injury was induced by 1 min of spinal cord (L2–L3) compression. Upper (early administration) (**a**,**b**). At 10 min after injury (A) (dashed bars), saline 5 mL/kg ip (white bars), or BPC 157 10 µg/kg ip (grey bars) were applied, and the effects were assessed at 1 min, 5 min, 15 min, and 20 min intervals (**a**). Spinal cord swelling was assessed relative to healthy values (100%). Spinal cord hematoma was assessed relative to a hematoma at 10 min following injury (100%) just before therapy application (**b**). Low (delayed administration) (**c**,**d**). At day 4 after injury, saline 5 mL/kg ig or BPC 157 10 ng/kg ig were applied, and the effects were assessed at 1 min, 5 min, 15 min, and 20 min intervals. Spinal cord swelling (**c**) and hematoma (**d**) were assessed relative to values at day 4 (100%), just before therapy application. Means ± SD, * *p* < 0.05, at least vs. corresponding control; ^#^ *p* < 0.05, at least vs. values at 10 min after injury (A) (100%).

Assessment employing a camera attached to a VMS-004 Discovery Deluxe USB microscope revealed that the BPC 157 therapy markedly impacted spinal cord injury hematoma and swelling in the early as well as in the advanced spinal injury course (Figure 4).

##### Early Post-Injury Course, Immediate Effect

**Figure 5:**
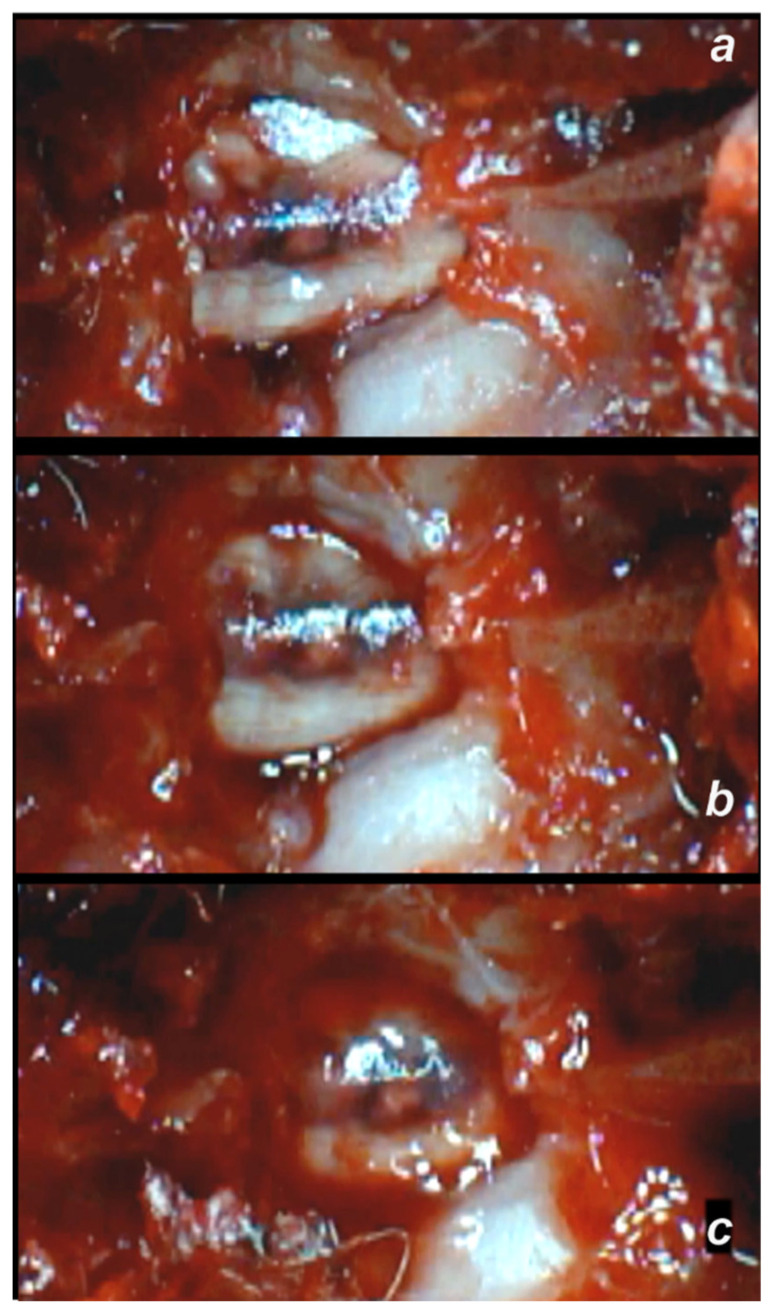
Spinal cord hematoma presentation following spinal cord compression (for 1 min) (a camera attached to a VMS-004 Discovery Deluxe USB microscope (Veho, Denver, CO, USA)). Presentation at 10 min following injury, before therapy saline (5 mL/kg ip) (**a**), and after saline application, at 10 min (**b**) and at 20 min (**c**).

(i). *Control.* The progressing course of the spinal cord swelling and hematoma (Figure 5a) could not be prevented by the saline intraperitoneal application at 10 min post-injury (Figure 5b,c).

**Figure 6:**
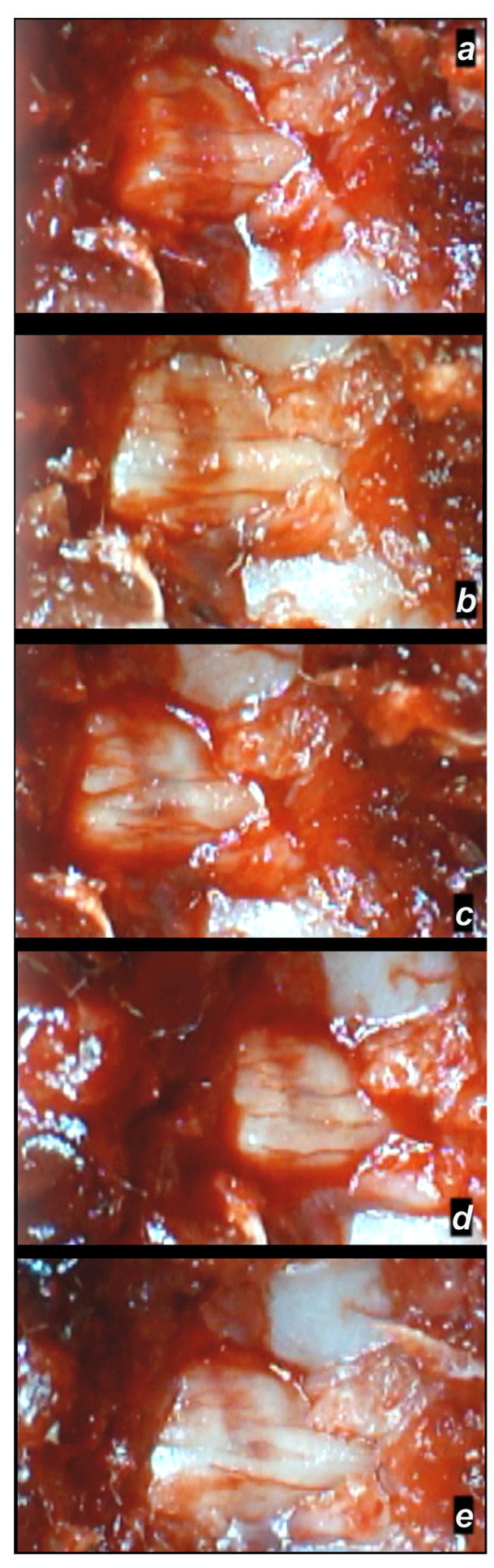
Spinal cord hematoma presentation following spinal cord compression (for 1 min) (a camera attached to a VMS-004 Discovery Deluxe USB microscope (Veho, Denver, CO, USA)). Presentation at 10 min following injury (**a**), before therapy, (**a**); and, (**b**–**e**) after BPC 157 application (10 µg/kg ip), immediately (**b**), at 5 min (**c**), at 10 min (**d**), and at 20 min (**e**).

*BPC 157.* Contrarily, the otherwise progressing course of the spinal cord swelling and hematoma (Figure 6a) was reversed by the intraperitoneal application of BPC 157 at 10 min post-injury, and the recovery occurred in minute intervals (Figure 6b–e).

##### Delayed Post-Injury Course, Immediate Effect

**Figure 7:**
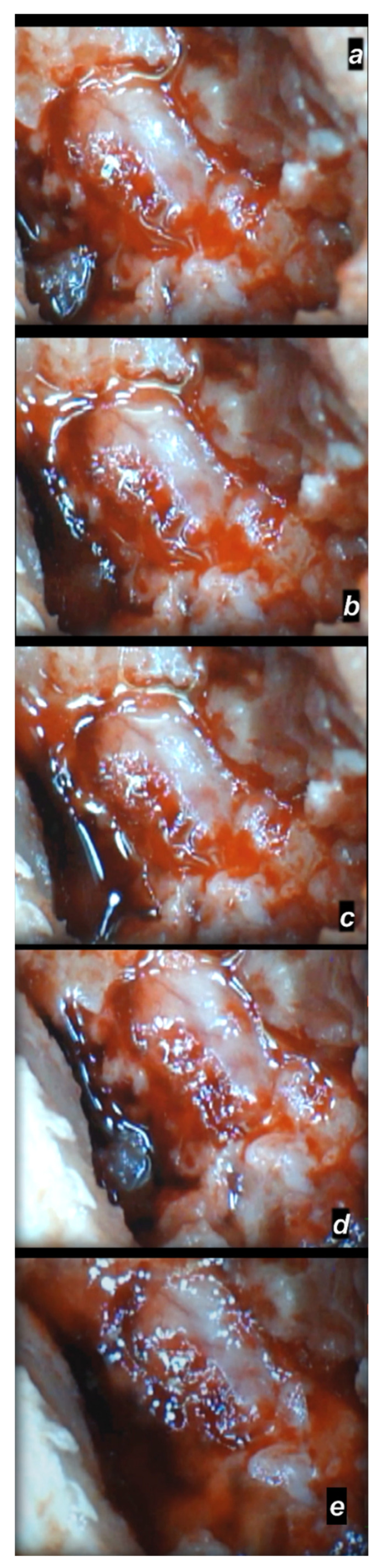
Spinal cord swelling and hematoma presentation following spinal cord compression (for 1 min), presentation at day 4 following injury (a camera attached to a VMS-004 Discovery Deluxe USB microscope (Veho, Denver, CO, USA)). Presentation before therapy (**a**), and, (**b**–**e**) after saline (5 mL/kg ig) application, immediately (**b**), at 5 min (**c**), at 10 min (**d**), and at 20 min (**e**).

(ii). *Control.* At day 4 post-injury, the advanced course of the spinal cord swelling and hematoma (Figure 7a) could be not prevented by the saline intragastric application as far could be seen during the 20 min post-therapy period (Figure 7b–e).

**Figure 8:**
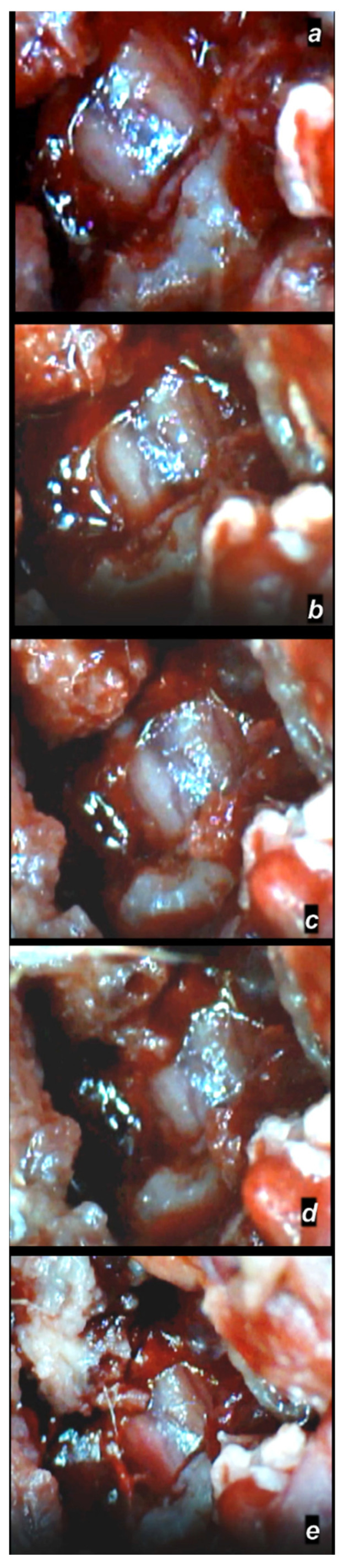
Spinal cord hematoma presentation following spinal cord compression (for 1 min), presentation at day 4 following injury (a camera attached to a VMS-004 Discovery Deluxe USB microscope (Veho, Denver, CO, USA)). Presentation before therapy (**a**), and, (**b**–**e**) after therapy with BPC 157 (10 µg/kg ig) application, immediately (**b**), at 5 min (**c**), at 10 min (**d**), and at 20 min (**e**).

*BPC 157.* Contrarily, the otherwise definitive course of the spinal cord swelling and hematoma (Figure 8a) at day 4 post-injury was reversed by the BPC 157 intragastric application, and the immediate effect of the recovery occurred in minute intervals (Figure 8b–e).

Thus, the control rats with a spinal cord injury had a persistent hematoma and persistent spinal cord swelling during both the early (10–30 min post-injury) and protracted (day 4) post-injury periods (Figure 4, Figure 5, and Figure 7). Contrarily, BPC 157 therapy rapidly attenuated spinal cord hematoma and swelling when given to rats with acute injury, as well as to rats with a protracted spinal cord injury (day 4 post-injury) (Figure 4, Figure 6, and Figure 8).

### 3.2. Microscopy

**Figure 9:**
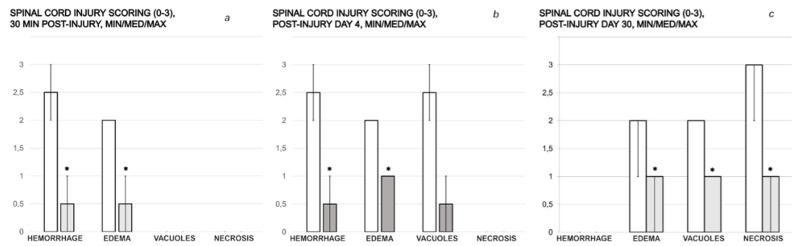
Spinal cord injury scoring (0–3), Min/Med/Max (hemorrhage, edema, vacuolation of tissue matter, necrosis) at the end of the experiment. Left (**a**). (i) One single intraperitoneal medication (saline 5 mL/kg b.w. (white bars); BPC 157 2 µg/kg b.w. (grey bars)) was administered at 10 min post-injury, and rats were sacrificed at 20 min thereafter (i.e., 30 min after injury). Middle (**b**). (ii) One single intragastric medication (saline 5 mL/kg b.w. (white bars); BPC 157 10 ng/kg b.w. (gray bars)) was administered at day 4 post-injury, and rats were sacrificed at 20 min thereafter. (iii) Right (**c**). Assessment at the post-injury day 30. In those rats who already had a definitive tail paralysis, the therapy began on day 4, and it was continuously given perorally in drinking water (water 12 mL/rat/day (white bars); BPC 157 10 μg/kg b.w., 0.16 μg/mL (gray bars)) until day 30 post-injury. * *p <* 0.05, at least vs. the corresponding control.

The microscopic assessment demonstrated a rapid beneficial effect of BPC 157 application (Figure 9) when applied soon after injury infliction, or when applied later in the rats with a protracted spinal cord injury (Figure 9).

#### 3.2.1. Early Post-Injury Course, Immediate Effect

**Figure 10:**
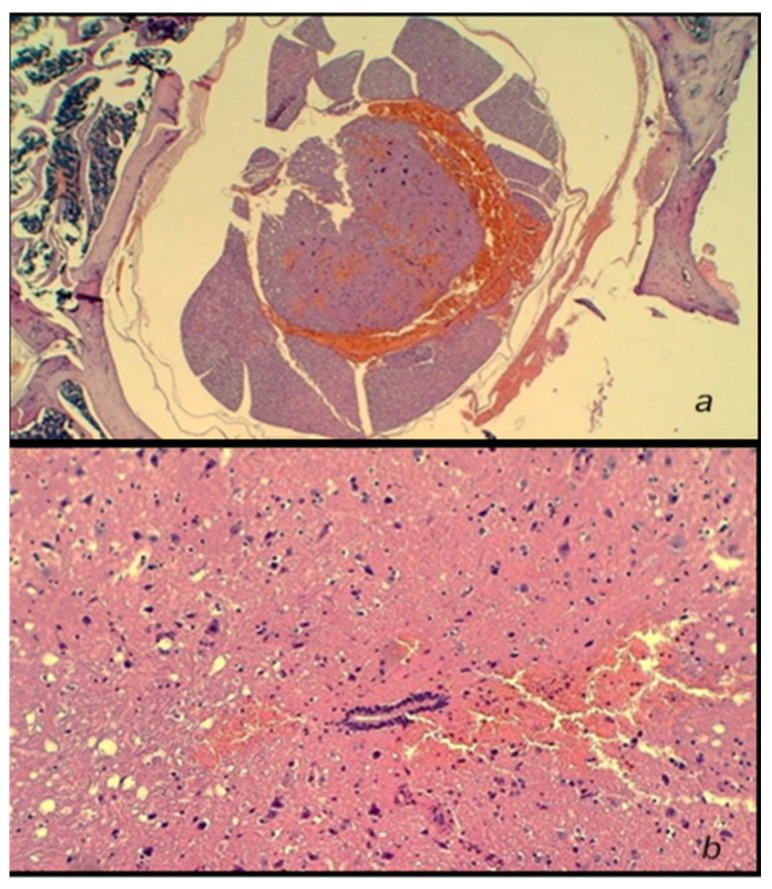
Neuropathological changes in the posttraumatic spinal cord and edema, and a massive hemorrhage at 10 min post-injury in the lateral and posterior white columns, as well as within the intermediate grey matter at the lesion site. HE staining; magnification 40× (upper) (**a**) and 200× (low) (**b**).

(i). At 10 min after injury, a pronounced hemorrhagic zone over the lateral and posterior white columns, as well as within the grey matter, was immediately present after piston removal in all rats (Figure 10).

*Control.* Over the next 20 min following saline administration, at 30 min post-injury an advanced and massive hemorrhage and edema in both areas were found in the control rats.

**Figure 11:**
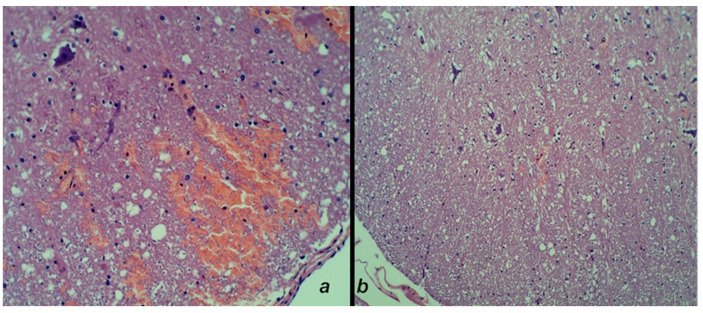
Post-injury time of 30 min-. Neuropathological changes in the posttraumatic spinal cord at the lesion site after 20 min following medication, which was applied at 10 min post-injury time. (**a**) The control group edema and massive hemorrhage in the area of the intermediate grey matter and the lateral and posterior white columns. (**b**) BPC 157 rats with minimal edema and hemorrhage (HE staining; magnification 100×).

*BPC 157.* Contrarily, in rats that received BPC 157 administration, only a discrete edema and a minimal hemorrhage were noted (Figure 11).

#### 3.2.2. Delayed Post-Injury Course, Immediate Effect

(ii). *Control.* At day 4, in the control animals, a large hemorrhagic zone was found predominantly over the lateral and posterior white columns, due to the consequent prominent edema and vacuolation of tissue matter which were obtained from the posterior and lateral spinal column tract of white matter, the anterior horn, and intermediate grey matter.

**Figure 12:**
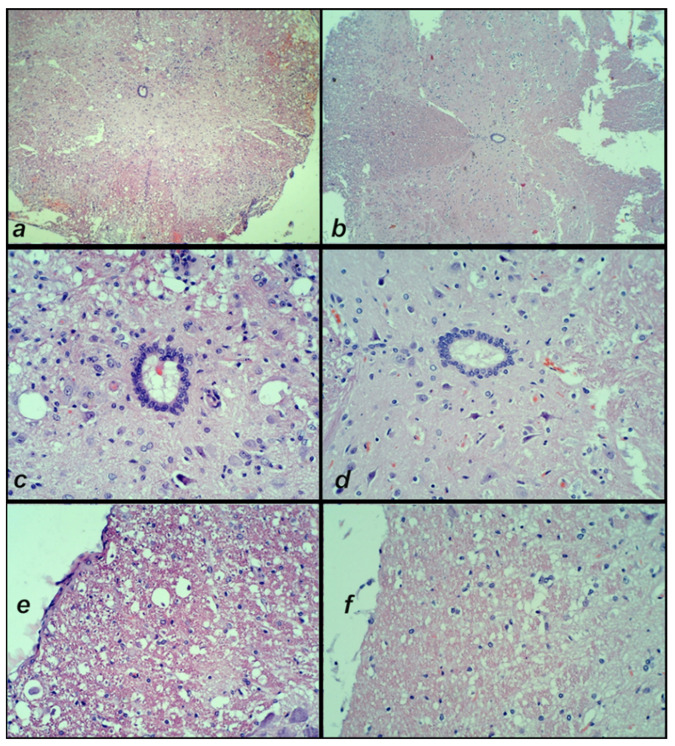
Post-injury day 4 at 20 min following the application of medication. Neuropathological changes in the posttraumatic spinal cord in the anterior horn and the intermediate grey matter at the lesion site. (**a**) Control rats with a large hemorrhagic area, (**c**,**e**) edema and confluent vacuolation of tissue matter in the anterior horn and intermediate grey matter. (**b**) Mild hemorrhage area in the lateral column and (**d**,**f**) the mild edema and discrete vacuolation of tissue matter in the anterior horn and intermediate grey matter in the BPC 157 rats. (HE staining; magnification 40× and 400×).

*BPC 157.* Contrarily, in the BPC 157 rats there was only a mild hemorrhage, and only a discrete vacuolation of tissue matter (Figure 12).

#### 3.2.3. Delayed Post-Injury Course, Long-Term Effect

(iii). *Control.* At day 30 post-injury, histochemical staining using Luxol fast blue showed a loss of myelin fibers of the posterior and lateral spinal column tracts, along with the anterior horn, and intermediate grey matter at the lesion site in the controls.

**Figure 13:**
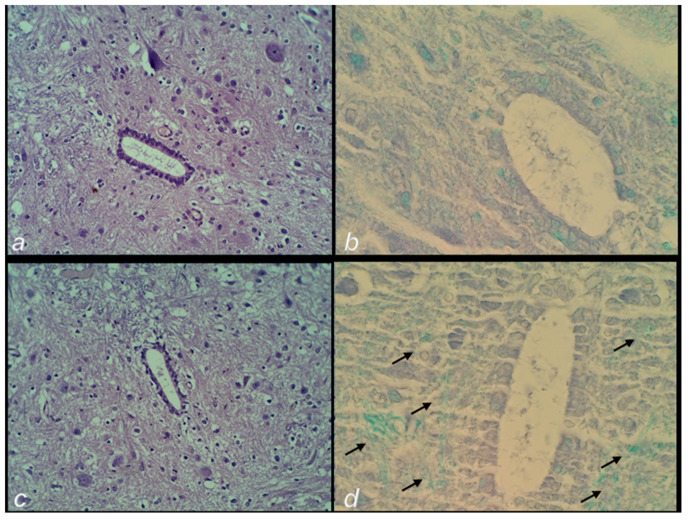
Post-injury day 30 with the therapy given since post-injury day 4. Neuropathological changes in the posttraumatic spinal cord in the intermediate grey matter at the lesion site. Control. Edema (**a**) and loss of neurons and loss of myelin fibers (**b**). BPC 157. Minimal edema (**c**) and with preserved myelin fibers (arrows) (**d**) (HE (**a**,**c**) and Luxol fast blue staining (**b**,**d**); magnification 400×).

**Figure 14:**
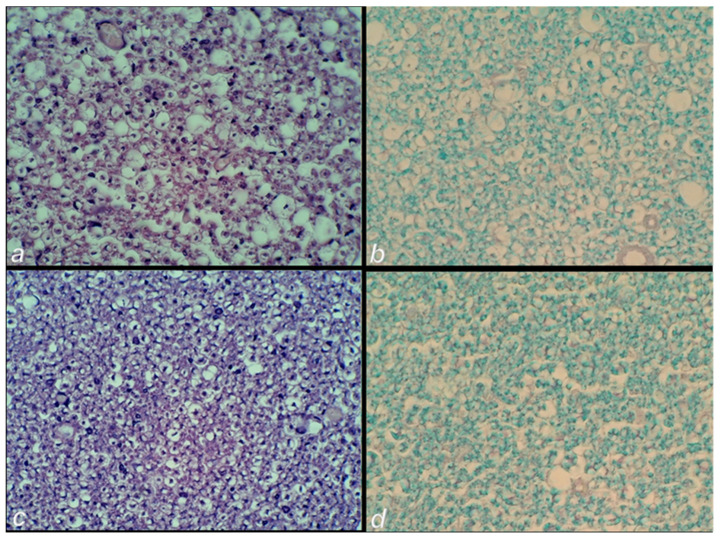
Post-injury day 30 with the therapy given since post-injury day 4. Neuropathological changes in the posttraumatic spinal cord in the lateral spinal column tract at the lesion site. (**a**) Control group edema and loss of neurons (**b**) loss of myelin fibers. (**c**) BPC 157 rats presented a minimal edema and (**d**) with preserved myelin fibers (HE (**a**,**c**) and Luxol fast blue staining (**b**,**d**); magnification 400×).

*BPC 157.* Contrarily, BPC 157 rats presented no demyelination process (Figure 13 and Figure 14).

### 3.3. mRNA Expression

**Figure 15:**
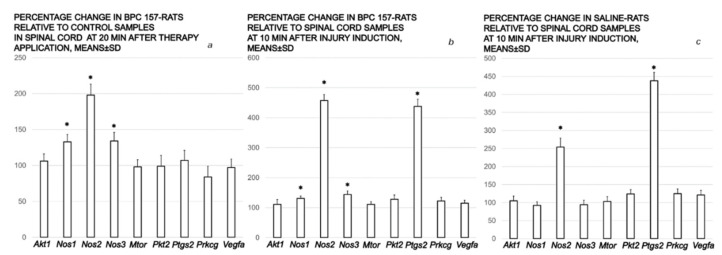
mRNA expression. Real-time PCR determination of mRNA expression of a set of targeted genes in the spinal cord samples. At 10 min after injury saline 5 mL/kg ip or BPC 157 10 µg/kg ip were applied, and the effects were assessed at 10 min after injury and 20 min after therapy application. Left (**a**). Results are expressed as percentage changes (Means ± SD) of BPC 157 10 µg/kg relative to the control samples at 20 min after therapy application. Middle (**b**). Results are expressed as percentage changes (Means ± SD) of BPC 157 10 µg/kg rats at 20 min after therapy application relative to the spinal cord samples at 10 min after injury. Right (**c**). Results are expressed as percentage changes (Means ± SD) of saline 5 mL/kg rats at 20 min after therapy application relative to the spinal cord samples at 10 min after injury. *p* < 0.05 is marked with *. Results without a * have no biological difference to the control samples.

Real-time PCR determination of mRNA expression of a set of targeted genes in the spinal cord samples presented no significant changes in *Akt1*, *Mtor*, *VEGFa*, *Prkcg*, and *Ptk2* values. At 10 min after injury, saline rats presented increased *Nos2* and *Ptgs2* expression, while BPC 157 rats presented with increased *Nos1*, *Nos2*, *Nos3*, and *Ptgs2* expression. At 30 min after injury, BPC 157-treated rats displayed increased *Nos1*, *Nos2*, and *Nos3* expression relative to the control animals (Figure 15).

**Figure 16:**
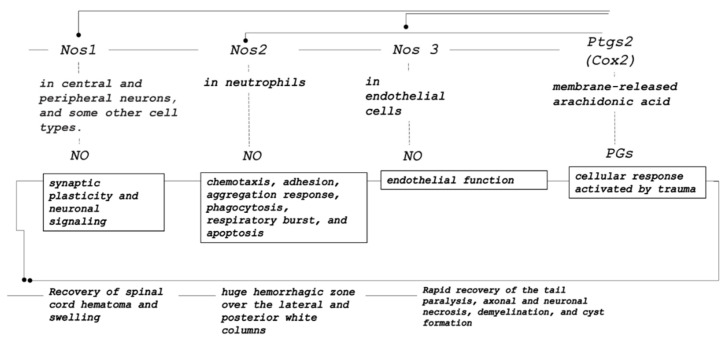
Illustration of the effects obtained in mRNA studies as the molecular background of the rapid efficacy of the BPC 157 therapy. We indicated that BPC 157 therapy should be specifically related to the NO system (increased *Nos 1*, *Nos 2*, *Nos3*) and prostaglandins system (increased *Ptgs2* (*Cox 2*)) pathways, since *Akt1*, *VEGFa*, *Mtor*, *Prkcg*, and *Pkt2* were not affected by BPC 157 therapy. In BPC 157 rats, the increase in *Nos 1*, *Nos 2*, and *Nos3* meant the synergistically increased capability of synaptic plasticity and neural signaling (*Nos1*), increased neutrophils function (i.e., chemotaxis, adhesion, aggregation response, phagocytosis), and endothelial function (*Nos 3*), synergistically affecting the prostaglandin system (*Cox 2*), and the cellular response activated by trauma. Together, these effects might be responsible for the rapid therapy effect in the spinal cord-injured rats.

In summary, after the point of the administration of BPC 157, the injury course definitively changed. The intraperitoneal as well as the intragastric application of BPC 157 achieved the immediate counteracting effect on the hematoma and swelling caused by spinal cord compression in rats. This beneficial effect equally occurred at an earlier post-injury time (i.e., 10 min) as well as at a later post-injury time (i.e., 4 days), likely accounting for much of the rapid and then the long-term sustained beneficial effects of BPC 157 therapy. The significant and persistent hematoma in the controls, as a regular consequence of the ischemic/reperfusion injury made by spinal cord compression, regressed after BPC 157 therapy application. The regression was macroscopically visible under a microcamera within a mere 20 min period. Microscopically, with application at 10 min following injury, the subsequent 20 min period (10 min–30 min post-injury) resulted in a regression to only a discrete edema and minimal hemorrhage at 30 min post-injury. This is supported by particular changes in the gene expression (i.e., increased *Nos1*, *Nos2*, and *Nos3* expression along with the increased *Ptgs2* expression) (Figure 16). Likewise, with application at day 4 post-injury, the subsequent 20 min period after therapy administration produced a similar regression to only mild hemorrhage and a discrete vacuolation of tissue matter. Thus, consequently, in the day 4–day 30 post-injury study, BPC 157 rats taking BPC 157 in drinking water rapidly presented with tail function recovery, and no demyelination (Luxol fast blue staining).

## 4. Discussion

To approach the spinal cord-injured rats that experienced the rapid and sustained recovery of BPC 157 therapy [1], spinal cord hematoma and swelling, we should note the diversity of the processes in the early and late course of spinal cord injury and the similar therapeutic effect. Possibly, this congruence might suggest an innate essential common point for the immediate events occurring following BPC 157 application at 10 min and at day 4 post-injury and the long-term beneficial outcome in either of those circumstances, unifying the function of BPC 157 in the early single intraperitoneal administration, the late term intragastric administration, and the continuous oral regimen. This would represent a certain innovation and practical value.

Of note, while all of the rats already presented the huge hemorrhagic zone over the lateral and posterior white columns, as shown at 10 min post-injury [1], in the rats with BPC 157 therapy, the spared white matter was a consistent finding in the injured spinal cord [1]. Since after spinal cord injury the hind limbs’ functional motor recovery requires salvaged white matter [81,82,83], this point closely correlates with the functional restoration of the paralyzed tail. Considering the presentation of the injuries in the grey matter on day 4 post-injury, and the counteracting effect of the BPC 157 application at that time, the immediate events occurring after BPC 157 application may involve both white and grey matter for this initial beneficial point to be demonstrated directly.

Following spinal cord compression injury, the subsequent administration of the BPC 157 stands in the reperfusion. The beneficial effect of this point is also directly documented in other models (i.e., [36,37,38,39,40,41,42,43,48,84,85]) and does not require any preconditioning in the ischemia period, an approach commonly used in other spinal cord injury studies [75,86,87]. To illustrate, BPC 157 application in reperfusion has particular effects. In the stroke rats, BPC 157 counteracted the neuronal damages in the brain, memory loss, and locomotion and coordination disturbances [48]. Hippocampal tissues showed the corresponding expression of the particular genes [48]. Resolution of the similar lesions carried out on vital organs (i.e., liver, lung, heart, gastrointestinal tract) (i.e., after clamping of the portal triad (Pringle maneuver) of the left colic artery and vein (ischemic/reperfusion colitis)) [36,85] has also been demonstrated.

Furthermore, the true therapeutic effect of BPC 157 therapy is likely different from the simple hemostatic effect that would as such attenuate spinal cord injury [68]. BPC 157 therapy evidently exhibited a particular hemostatic effect in rats [38,69,70,71]. Namely, it goes with the maintained thrombocyte function [70] and has no effect on coagulation factors [70,71,72]. Likewise, it goes with the particular cytoprotective effect on endothelium maintenance (shown to be active already within minutes in the stomach cytoprotection studies) and functioning in the Virchow triad circumstances (for a review, see [10,18]) (i.e., rapid activation of the alternative bypassing (collateral) pathways [36,37,38,39,40,41,42,43,48,85]). Consequently, BPC 157 both prevented and reversed thrombosis formation after abdominal aorta anastomosis [84] and major artery and/or vein occlusion [36,37,38,39,40,41,42], or general vascular failure in occlusion-like syndromes (i.e., major intoxication with alcohol and lithium, which are endothelium damaging agents, maintained severe intra-abdominal hypertension and myocardial infarction [43,44,45,46]). As a follow up, BPC 157 alleviated peripheral and central vascular occlusion disturbances [36,37,38,39,40,41,42], in both ischemia and reperfusion [36,48,85]. As it was the case in the spinal cord-injured rats, once the therapeutic effect started in the rats with the permanently occluded vessel(s), neither of the ligation/occlusion induced-disturbances reappeared. There is a constant therapeutic effect that could adequately compensate all harm from ligation (occlusion) [36,37,38,39,40,41,42]. Likewise, reestablished blood flow may certainly contribute to the rapid recovery effect noted in the spinal cord-injured rats.

In addition, we can take the spinal cord compression injury and BPC 157 as a part of its wound-healing effect (i.e., the wound healing process accomplished all four major events: vascular constriction, loose platelet plug, fibrin mesh to ensure the stability of platelet plug, and dissolution of the clot) (for a review, see, i.e., [18,19]). Consequently, BPC 157 may counteract prolonged bleeding at the site of spinal cord compression injury by its wound healing effect as well. This occurred in the various models, i.e., amputation of the tail, leg [70,71], prolonged occlusion of the vein [38], anticoagulants or aspirin [70,71] or L-arginine (NOS-substrate, NO-system over-activity) [71] application, and organ perforation [69]. This extensive beneficial effect may be of practical importance. Namely, spinal epidural and subdural hematomas most often occur in patients who are on anticoagulants [46], while, as mentioned, BPC 157 strongly maintains platelet function and counteracts thrombocytopenia, while mitigating anticoagulant-induced prolonged bleeding, in particular [69,70,71,72].

Together, given the rapid onset of the tail paralysis, and the onset of the therapy effect, these may support the resolution of the rapid hematoma, and spinal cord decompression as the essential effects of BPC 157 application, at both the very early post-injury time (10 min (post-injury)–30 min (therapy)) and the later post-injury time point (day 4).

As such, the beneficial effect of BPC 157 is a particular one, and its immediate presentation in the spinal cord-injured rats in the primary injury (10 min post-injury) and in secondary injury (post-injury day 4) should reflect its molecular background. These should be perceived along with the earlier findings on the molecular mechanisms of neuroprotection in the experimental spinal cord injury and the therapeutic potential of pharmacological treatments in spinal cord injury [88,89]. Here, mRNA expression studies in the spinal cord in the prime injury-injured tissue showed a specific effect (note, this phase starts at the time of the trauma and lasts about 2 h) [90]. *Cox2* and *Nos 2* increased in both controls and BPC 157 rats, *Nos 1*, *Nos 2*, and *Nos3* increased in BPC 157 rats relative to controls, and *Akt1*, *VEGFa*, *Mtor*, *Prkcg*, and *Pkt2* were not affected. This effect seems to be specific for the spinal cord injury moment, providing that in the recovery of blood flow in the ischemic muscle, BPC 157 is associated with VEGFR2 activation and up-regulation [58]. Thereby, in the prime injury, as a decisive determinant of injury severity and future disability [88,89], these particular findings of the mRNA expression studies in the spinal cord injured tissue, show the immediate specific effect of NO/prostaglandins in BPC 157 therapy. This should be seen in the general terms of the acute spinal cord injury, as the traumatic primary injury as the epicenter of the lesion triggers all pathological sequele, and comprises cell death, local severance of axons, and damage to blood vessels with the focal hemorrhage around the injury site [88,89]. This might be a further complex cascade of molecular events termed ‘secondary injury’, and progressive degeneration ranging from early neuronal apoptosis at the lesion site to delayed degeneration of intact white matter tracts, and, ultimately, expansion of the initial injury [88,89]. Most probably, given the relatively immediate effects BPC 157 therapy, its molecular pathways are not related to two major pro-inflammatory transcription factors activated in inflammation [91,92]: the activator protein-1 (AP-1) and nuclear factor kappa B (NF-κB). Namely, their binding activity increased later after spinal cord injury (i.e., AP-1 at 1 h, peaking at 8 h, [93], NF-κB peaked between 1- and 3-days post-injury [94]). Western blot analysis revealed that tumor necrosis factor-alpha (TNF-α) expression began at 4 h post-injury and peaked at 24 h [94], and cytosolic phospholipase A2 (cPA2) peaked at 4 h after injury [95]. Finally, given the immediate effects of BPC 157 therapy as a direct effect in reperfusion, its molecular pathways might be not related to the pathways considered with the agents that need preconditioning [85,86]. As an effect not limited to the method of application (providing useful parenteral, peroral), the background of BPC 157 might not be related to the relations of the agents that required implanted capsules (testosterone, estradiol) [96]. Most likely, considering the described effects of BPC 157, and its specific effect on NO/prostaglandins that occurred rapidly, it might be distinctive from the relations of the phosphatase and tensin homologue (PTEN) inhibitor, the bisperoxovanadium activation of PI3K/AKT-mTOR signaling [97], or the methylprednisolone inhibition of TNF-alpha expression and NF-kB activation [94]. Thus, this BPC 157 NO/prostaglandins effect may be a precisely coordinated series of events related to the effect of the BPC 157 therapy. It may be hypothesized that COX-2 products may have a protective role (i.e., explaining the lack of efficacy of COX-2 inhibition [98]), which is in fact a spontaneous response against injury [99]. After stimulation, action through the NO system shows the high output of NO from iNOS acting in antimicrobial, antiviral, antiparasitic and tumoricidal processes and the cytotoxic effect of NO involved in immunological and tissue-damaging actions (for a review, see [100]). NO from iNOS modulates chemotaxis, adhesion, aggregation response, phagocytosis, respiratory burst, and apoptosis in neutrophils through cGMP-dependent and independent pathways [101]. COX-2 also has an important role in various inflammatory and induced settings, acting through prostaglandin-mediated systems [102]. Because they are not stored, prostaglandins are synthesized de novo from membrane-released arachidonic acid, indicating a cellular response activated by trauma [102]. In addition to the prostaglandins system [14], BPC 157 may also act beneficially through the NO system (*Nos 1*, *Nos 2*, *Nos3*) (for a review, see [13]). It may also favor the high output of NO from the neuronal NO synthase (nNOS) as the up-regulation of the nNOS in resident spinal cord cells [102]. Possibly, with respect to the underlying disease (which was markedly counteracted in BPC 157 rats), more activity of *eNOS* (the constitutive isoforms instantly increase along with COX2), and more activity of *nNOS* (NO produced by nNOS is thought to mediate synaptic plasticity and neuronal signaling) [103,104], may be intrinsic effects of BPC 157. In support, increased *Nos 1*, *Nos 2*, and *Nos3* might act synergistically with BPC 157 since, as shown before, there was a particular modulatory effect of BPC 157 on the functions of the NO system (for a review, see [13]). Illustratively, BPC 157 induced the release of NO on its own, which was quite resistant to the NOS blocker L-NAME, counteracted the adverse effect of both the NO blockade and NO over-stimulation in many models and species (for a review, see [13]), and interacted with NO-specific molecular pathways [54,58] (i.e., BPC 157 regulates vasomotor tone and the activation of the Src-Caveolin-1-endothelial nitric oxide synthase pathway [54]). Likewise, BPC 157 might maintain the functioning of the prostaglandins system by counteracting the adverse effect of various NSAIDs (for a review, see [14]). Finally, both beneficial effects, early and prolonged, consistently noted, might eliminate the possible harmful later effects (reperfusion, free radical formation) [93]. Moreover, BPC 157 induces a free radical scavenger effect [4,60,61,62]. Thus, we may suggest an adequate capacity of the oxidant defense system to counteract possible oxidative stress [4,60,61,62]. Note, similar effects appeared in another bleeding study (i.e., perforated stomach) as well [105].

It is likely that this effect may be responsible for the beneficial effect and recovery seen with both the early and delayed therapy. This might occur regardless of the possible limitation of the results only reflecting mRNA levels, which may not correlate with protein levels [48]. This may also be the case in the consideration of the inherent limitation of the animal models [106]. Illustratively, midthoracic injuries, various degrees of hindlimb paralysis, and trunk instability in quadrupeds would have significantly different behavioral outcomes than what can occur in bipeds such as humans [107]. On the other hand, the originally chosen model [108] that we adopted [1,108], laminectomy at the lumbar level L2–L3, which corresponds to the sacrocaudal spinal cord S2-Co1, as the particular site of the injury in rats [108], might avoid most of limitations of the other spinal cord injury models [1,106,108]. It forwarded a particular relevance of sacral spinal rats, as the symptoms of spasticity in the tail muscles correspond fairly with those seen in the limb muscles of humans with a spinal cord injury [108]. Illustratively, the compression force of 60–66 g used produced a severe injury [1,109], and all of the therapy effects may provide relevant information for the human disturbances as well. Thus, previous studies [1] and present findings indicate a strong correlation (functional, macroscopic, microscopic, and electrophysiologic recovery, gene analysis, evident upon administration, immediately, daily, weekly, monthly, and one year) between the two regimens of BPC 157 application (early vs. delayed). From a practical viewpoint of the spinal cord injury, the important findings highlighted the rapid recovery of the tail paralysis, axonal and neuronal necrosis, demyelination, and cyst formation [1], which were counteracted as result of the delayed therapy that was given per orally in drinking water. Furthermore, there were the immediate findings obtained after the one-time application administered at the earliest point 10 min after injury and at the postponed point of the definitive injury at day 4 after injury infliction. Together, in the spinal cord-injured rats, these consistent effects of BPC 157 therapy demonstrated the full practical importance (i.e., wide ranging ability to achieve the appropriate beneficial outcome after spinal cord injury in rats (i.e., bolus (intraperitoneal, intragastric)) vs. small amounts taken ad libitum (therapy given in the drinking water)). These should be seen with the evidence that in addition to being isolated from human gastric juice, BPC 157 was found in in situ hybridization and immunostaining studies in humans to be largely distributed in tissues [6] and may have additional physiologic regulatory roles that may be fully implemented in its profound healing capacity. A particular advantage for revealing this concept (i.e., BPC 157 therapy, a native cytoprotective gastric pentadecapeptide, not degraded in human gastric juice, safe (lethal dose (LD1) not achieved), no adverse effect in clinical trials (i.e., ulcerative colitis) (for a review, see [6,18,19])) in practice, and in the spinal cord injury, in particular, is its very safe profile. The fact that an LD1 was not achieved is a point recently confirmed in a large study by Xu and collaborators [110].

## Figures and Tables

**Table 1:** Selected genes and TaqMan Assay specifications.

Gene Symbol	Synonyms	Gene Name	TaqMan Assay ID	NCBI Reference Sequence	Amplicon Length (bp)
*Actb*		Actin, beta	Rn00667869_m1	NM_031144.3	91
*Akt1*	PKB, RAC	AKT serine/threonine kinase 1	Rn00583646_m1	NM_033230.2	87
*Nos1*	nNOS, bNOS	Nitric oxide synthase 1	Rn00583793_m1	NM_052799.1	65
*Nos2*	iNos, Nos2a	Nitric oxide synthase 2	Rn00561646_m1	NM_012611.3	77
*Nos3*	cNOS, eNos	Nitric oxide synthase 3	Rn02132634_s1	NM_021838.2	117
Mtor	Frap1, RAFT1, RAPT1	Mechanistic target of rapamycin kinase	Rn00693900_m1	NM_019906.1	70
*Prkcg*	PKC, Prkc	Protein kinase C, gamma	Rn00440861_m1	NM_012628.1	89
*Ptgs2*	COX-2, Cox2, cyclooxygenase 2	Prostaglandin-endoperoxide synthase 2	Rn01483828_m1	NM_017232.3	112
*Ptk2*	FAK, FADK1, FRNK	Protein tyrosine kinase 2	Rn01505115_m1	NM_013081.2	81
*Vegfa*	VEGF-A, VPF	Vascular endothelial growth factor A	Rn01511601_m1	NM_001110333.2	69
